# Transcatheter Aortic Valve Implantation in Japanese Patients With Large Annulus: The OCEAN-TAVI Registry

**DOI:** 10.1016/j.jacasi.2024.07.002

**Published:** 2024-08-13

**Authors:** Kyohei Onishi, Kazuki Mizutani, Kosuke Fujita, Masafumi Ueno, Genichi Sakaguchi, Gaku Nakazawa, Yohei Ohno, Fumiaki Yashima, Toru Naganuma, Norio Tada, Shinichi Shirai, Futoshi Yamanaka, Masahiko Noguchi, Masaki Izumo, Kensuke Takagi, Masahiko Asami, Hiroshi Ueno, Hidetaka Nishina, Toshiaki Otsuka, Yusuke Watanabe, Masanori Yamamoto, Kentaro Hayashida

**Affiliations:** a Division of Cardiology, Department of Medicine, Kindai University Faculty of Medicine, Osaka, Japan; b Division of Cardiovascular Surgery, Department of Surgery, Kindai University Faculty of Medicine, Osaka, Japan; c Department of Cardiology, Tokai University School of Medicine, Isehara, Japan; d Department of Cardiology, Saiseikai Utsunomiya Hospital, Tochigi, Japan; e Department of Cardiology, New Tokyo Hospital, Matsudo, Japan; f Department of Cardiology, Sendai Kosei Hospital, Sendai, Japan; g Department of Cardiology, Kokura Memorial Hospital, Kokura, Japan; h Department of Cardiology, Shonan Kamakura General Hospital, Kamakura, Japan; i Department of Cardiology, Tokyo Bay Urayasu Ichikawa Medical Center, Urayasu, Japan; j Department of Cardiology, St Marianna University, Kanagawa, Japan; k Department of Cardiology, National Cerebral and Cardiovascular Center, Osaka, Japan; l Division of Cardiology, Mitsui Memorial Hospital, Tokyo, Japan; m Department of Cardiology, Toyama University Hospital, Toyama, Japan; n Department of Cardiology, Tsukuba Medical Center Hospital, Tsukuba, Japan; o Department of Hygiene and Public Health, Nippon Medical School, Tokyo, Japan; Center for Clinical Research, Nippon Medical School Hospital, Tokyo, Japan; p Division of Cardiology, Teikyo University Hospital, Tokyo, Japan; q Department of Cardiology, Toyohashi Heart Center, Toyohashi, Japan; r Department of Cardiology, Nagoya Heart Center, Toyohashi, Japan; s Department of Cardiology, Gifu Heart Center, Toyohashi, Japan; t Division of Cardiology, Keio University School of Medicine, Tokyo, Japan

**Keywords:** aortic stenosis, paravalvular leakage, prosthesis-patient mismatch, transcatheter aortic valve implantation

## Abstract

**Background:**

East Asians have smaller aortic valve complexes than individuals from Western countries, and few studies have reported transcatheter aortic valve implantation (TAVI) outcomes in Asian patients with a large annulus.

**Objectives:**

This study aimed to compare the short- and long-term outcomes of TAVI using balloon-expandable valves (BEVs) and self-expandable valves (SEVs) in Asian patients with a large annulus.

**Methods:**

The study retrospectively analyzed the data from the OCEAN-TAVI (Optimized Transcatheter Valvular Intervention Transcatheter Aortic Valve Implantation) registry. A large annulus was defined by an annular area ≥500 mm^2^ and an average diameter ≥25 mm as measured by computed tomography. The primary endpoint was 3-year all-cause mortality. Secondary endpoints were 3-year heart failure rehospitalization (HFR) after TAVI, short-term outcomes of TAVI, and changes in valve function 2 years after TAVI.

**Results:**

Among 773 patients, 671 underwent BEV TAVI. The SEV TAVI group showed a significantly higher incidence of greater than moderate paravalvular leakage (PVL) (*P* < 0.001), and an increased pacemaker implantation rate (*P* = 0.035). The incidence of prosthesis-patient mismatch did not differ between the 2 groups. The Kaplan-Meier curve showed no significant differences in 3-year all-cause mortality and HFR rates (log-rank *P* = 0.900), and echocardiographic valve function at 2 years post-TAVI did not differ between the 2 groups.

**Conclusions:**

The lack of differences in postoperative valve performance and long-term prognosis between BEV TAVI and SEV TAVI highlights the importance of selecting valves that can reduce the pacemaker implantation rate and PVL grade in the acute phase in patients with a large annulus. (Optimized Transcatheter Valvular Intervention Transcatheter Aortic Valve Implantation [OCEAN-TAVI]; UMIN000020423)

Transcatheter aortic valve implantation (TAVI) is a comparable treatment option to surgical aortic valve replacement for patients with aortic stenosis, regardless of the patient’s risk level, and it has been shown to provide satisfactory short- and long-term clinical outcomes.^1^, ^2^, ^3^, ^4^, ^5^, ^6^ In particular, TAVI is more advantageous than surgical aortic valve replacement in patients with a small annulus and is associated with a lower incidence of prosthesis-patient mismatch (PPM).^7^ Furthermore, several studies have reported that TAVI with a self-expandable valve (SEV) is superior to TAVI with a balloon-expandable valve (BEV) in terms of the incidence of PPM in patients with a small annulus, whereas the incidence of pacemaker implantation (PMI) is higher in patients undergoing SEV TAVI than in patients undergoing BEV TAVI.^8^^,^^9^ However, a few studies that focused on patients with a large annulus found no significant difference in postoperative hemodynamics between BEV TAVI and SEV TAVI, but these investigators observed higher rates of significant paravalvular leakage (PVL) and valve embolization in SEV TAVI.^9^, ^10^, ^11^, ^12^

Nevertheless, the definition of a large annulus in these studies was ambiguous, and many studies focused on short-term results, whereas those studies evaluating long-term prognoses had an observation period of approximately 2 years.^9^, ^10^, ^11^, ^12^ Furthermore, many East Asians have smaller aortic valve complexes than individuals from Western countries,^13^ and we have previously reported that approximately one-fourth of all Japanese individuals have an average annulus diameter of ≤23 mm.^14^ These aspects highlight the need to report the short- and long-term results of BEV TAVI and SEV TAVI using large valves, especially in East Asian patients. Therefore, we investigated the short-term and 3-year postoperative clinical outcomes of BEV TAVI and SEV TAVI with large valves by using a large multicenter registry.

## Methods

### Study group

This analysis was performed retrospectively using data from the OCEAN-TAVI (Optimized Transcatheter Valvular Intervention Transcatheter Aortic Valve Implantation) registry, a prospective multicenter observational registry that assesses the real-world clinical outcomes of TAVI procedures conducted at 20 hospitals located in Japan.^15^ This registry was approved by the International Committee of Medical Journal Editors and is registered with the University Hospital Medical Information Network Clinical Trials Registry (UMIN000020423). The study protocol was developed in accordance with the Declaration of Helsinki and approved by the Ethics Committee of each participating hospital. All the patients provided informed consent before participating in the study. From October 2013 to December 2019, 7,393 patients who underwent TAVI were included in the registry. After excluding cases with missing computed tomography (CT) data before TAVI, missing echocardiography data at discharge, conversion to surgical aortic valve replacement, and valve-in-valve procedures, as well as patients who did not undergo transcatheter heart valve implantation, 773 patients with a large annulus were included in this analysis.

### Definition of a large annulus

A large annulus was defined by an annular area of ≥500 mm^2^ and an average diameter of ≥25 mm on CT measurements, thus necessitating a BEV with a diameter >26 mm or an SEV with a diameter >29 mm.

### Data collection

All the data were collected from the OCEAN-TAVI database. During the study period, Edwards SAPIEN-XT and SAPIEN-3 (Edwards Lifesciences) balloon-expandable prostheses and the Medtronic CoreValve, Evolut-R, and Evolut-Pro System (Medtronic) self-expandable prostheses were available in Japan. The local heart team members evaluated the indications for TAVI and made the final decision regarding the approach for TAVI.

### Endpoints

The primary endpoint of this study was 3-year all-cause mortality. The secondary endpoints were as follows: 3-year heart failure rehospitalization (HFR) after TAVI; short-term outcomes of TAVI, including the incidence of major vascular injury, coronary occlusion, aortic root rupture, need for a second valve, conversion to open surgery for repair, permanent PMI, ischemic stroke, acute kidney injury, and in-hospital death; technical success; device success on the basis of the definitions of the Valve Academic Research Consortium 2 criteria,^16^ and changes in valve function on echocardiography 2 years after TAVI.

### Statistical analyses

Continuous variables were presented as median and IQR (Q1-Q3), and categorical variables were presented as numbers and percentages. The study group was divided into BEV TAVI and SEV TAVI groups. The incidence of each 3-year endpoint was estimated using the Kaplan-Meier method and reported with 95% CIs. Differences between the groups were evaluated using log-rank tests. Differences in continuous and categorical variables among the groups were compared using Wilcoxon rank sum and chi-square tests, respectively. Statistical analyses were performed using R software version 3.6.3 (R Development Core Team). Statistical significance was set at *P* < 0.05.

## Results

Of the 773 patients, 671 (86.8%) underwent BEV TAVI. Patient characteristics are listed in Table 1. The median age of the entire cohort was 83 years, and 668 (86.4%) of the patients were male. The median Canadian Study of Health and Aging Clinical Frailty Scale score was 3, and the median Society of Thoracic Surgeons score was 5.1%. The median annulus area, diameter, and perimeter were 540.0 mm^2^ (range: 517.3-571.2 mm^2^), 26.2 mm (range: 25.7-27.0 mm), and 83.8 mm (range: 81.9-86.0 mm), respectively. The SEV TAVI group showed slightly worse baseline hemodynamic characteristics of aortic stenosis in echocardiographic assessments and a slightly higher rate of moderate to severe left ventricular outflow tract calcification, although the patients in the BEV TAVI group had a larger aortic annular size. Information on the TAVI procedure and periprocedural complications is presented in Table 2.

**Table 1:** Patient Characteristics

	Total (N = 773)	Balloon-Expandable Valve (n = 671)	Self-Expandable Valve (n = 102)	*P* Value
Age, y	86 (82-89)	83 (79-86)	84 (80-87)	0.422
Male	184 (14.7)	585 (87.2)	83 (81.4)	0.110
BSA, m^2^	1.40 (1.31-1.50)	1.60 (1.50-1.70)	1.59 (1.40-1.70)	0.003
BMI, kg/m^2^	21.8 (19.6-24.4)	22.8 (20.5-24.9)	22.3 (19.5-24.2)	0.035
Atherosclerotic risks				
Hypertension	1,004 (80.0)	536 (79.9)	81 (79.4)	0.912
Dyslipidemia	673 (53.6)	354 (52.8)	49 (48.0)	0.374
Diabetes mellitus	365 (29.1)	219 (32.6)	31 (30.4)	0.652
Current smoking	189 (15.2)	9 (1.4)	1 (1.0)	0.776
Atrial fibrillation	246 (19.6)	186 (27.7)	26 (25.5)	0.638
Pacemaker implantation	63 (5.0)	—	—	—
Previous coronary artery bypass graft	50 (6.4)	41 (6.7)	4 (2.9)	0.142
Previous PCI	213 (17.0)	192 (28.6)	24 (23.5)	0.286
Previous myocardial infarction	46 (3.7)	41 (6.1)	4 (3.9)	0.379
Coronary artery disease	396 (31.6)	—	—	—
Previous ischemic stroke	125 (10.0)	94 (14.0)	13 (12.7)	0.731
Peripheral artery disease	151 (12.1)	74 (11.0)	13 (12.7)	0.609
Cerebrovascular disease	128 (10.2)	—	—	—
Pulmonary disease	108 (8.6)	—	—	—
NYHA functional class				0.184
II	773 (61.7)	346 (51.6)	56 (54.9)	
III	291 (23.2)	218 (32.5)	25 (24.5)	
IV	77 (6.2)	51 (7.6)	13 (12.7)	
Clinical frailty scale	4 (3-4)	3 (3-4)	4 (3-4)	0.400
Laboratory data on admission				
eGFR, mL/min/1.73 m^2^	50.3 (39.3-63.3)	52.1 (39.4-64.7)	52.5 (43.6-63.0)	0.862
BNP, pg/mL	145.6 (65.8-343.2)	275.5 (118.8-604.9)	356.4 (167.1-657.0)	0.127
TTE data before TAVI				
LVEF, %	64.7 (58.9-69.0)	55.5 (44.7-63.7)	57.5 (40.5-66.0)	0.873
Mean AVPG, mm Hg	42.0 (34.7-52.8)	42.9 (33.6-56.0)	47.2 (36.1-65.2)	0.014
Peak AVPG, mm Hg	72.3 (64.0-89.1)	74.0 (60.5-92.2)	83.2 (64.1-107.8)	0.007
AVA with Doppler, cm^2^	0.66 (0.53-0.78)	0.71 (0.60-0.83)	0.60 (0.48-0.74)	<0.001
Index AVA, cm^2^/m^2^	0.46 (0.38-0.55)	0.44 (0.37-0.52)	0.38 (0.30-0.48)	<0.001
Bicuspid valve	20 (1.6)	78 (11.7)	18 (18.0)	0.075
CT data before TAVI				
Annulus area, mm^2^	367.0 (337.6-397.6)	544.0 (518.8-575.1)	524.6 (512.8-541.0)	<0.001
Mean annular diameter, mm	21.6 (20.7-22.5)	26.3 (25.7-27.1)	25.9 (25.6-26.3)	<0.001
Annular perimeter, mm	69.5 (66.6-72.4)	84.0 (82.0-86.4)	82.3 (81.5-84.0)	<0.001
Moderate or severe LVOT calcification	73 (6.2)	22 (3.4)	12 (12.0)	<0.001
STS score	6.0 (4.1-9.5)	5.0 (3.3-8.1)	5.4 (4.0-7.7)	0.275

Values are n (%) or median (Q1-Q3).

AVA = aortic valve area; AVPG = aortic valve pressure gradient; BMI = body mass index; BNP = B-type natriuretic peptide; BSA = body surface area; CT = computed tomography; eGFR = estimated glomerular filtration rate; LVEF = left ventricular ejection fraction; LVOT = left ventricular outflow tract; PCI = percutaneous coronary intervention; STS = The Society of Thoracic Surgeons; TAVI = transcatheter aortic valve implantation; TTE = transthoracic echocardiography.

**Table 2:** Procedural and Periprocedural Data

	Total (N = 773)	Balloon-Expandable Valve (n = 671)	Self-Expandable Valve (n = 102)	*P* Value
Procedural data				
Femoral approach	713 (92.4)	623 (93.0)	90 (88.2)	<0.001
Local anesthesia	269 (34.8)	221 (32.9)	48 (47.1)	0.005
Puncture	610 (80.3)	525 (79.5)	85 (85.0)	0.202
Valve type				—
Edwards SAPIEN XT	99 (12.8)	99 (14.8)	—	
Edwards SAPIEN 3	572 (74.0)	572 (85.2)	—	
Medtronic CoreValve	8 (1.0)	—	8 (7.8)	
Medtronic Evolut R	55 (7.1)	—	55 (53.9)	
Medtronic Evolut Pro	39 (5.1)	—	39 (38.2)	
Valve size, mm	29 (26-29)	29 (26-29)	29 (29-29)	<0.001
Procedure time, min	63 (49-85)	62 (47-82)	75 (61-99)	<0.001
TTE data after TAVI				
Mean AVPG, mm Hg	9.0 (7.0-12.0)	9.0 (7.0-12.0)	8.7 (6.0-11.1)	0.061
Peak AVPG, mm Hg	18.1 (14.4-23.0)	18.5 (14.4-23.0)	16.0 (12.3-22.8)	0.035
Effective orifice area index, cm^2^/m^2^	1.22 (1.04-1.43)	1.23 (1.04-1.44)	1.20 (0.97-1.35)	0.066
PVL grade moderate or greater	22 (2.9)	14 (2.1)	8 (7.8)	<0.001
Moderate PPM	49 (6.4)	40 (6.0)	9 (8.8)	0.269
Severe PPM	11 (1.4)	9 (1.3)	2 (2.0)	0.623
Periprocedural complications				
Major vascular complication	19 (2.5)	17 (2.5)	2 (2.0)	0.728
Coronary occlusion	7 (0.9)	6 (0.9)	1 (1.0)	0.932
Aortic root rupture	1 (0.1)	1 (0.1)	0 (0.0)	0.696
Second valve	5 (0.7)	3 (0.4)	2 (2.0)	0.076
Conversion to open surgery	2 (0.3)	2 (0.3)	0 (0.0)	0.581
Permanent pacemaker implantation	53 (6.9)	41 (6.1)	12 (11.8)	0.035
New onset of CLBBB	150 (21.3)	125 (20.4)	25 (27.5)	0.124
New onset of AF	27 (3.5)	24 (3.6)	3 (3.0)	0.753
Ischemic stroke	20 (2.6)	15 (2.2)	5 (4.9)	0.114
Acute kidney injury	74 (9.6)	64 (9.5)	10 (9.8)	0.932
In-hospital death	7 (0.9)	7 (1.0)	0 (0.0)	0.300
Technical success	749 (97.0)	650 (96.9)	99 (97.0)	0.918
Device success	708 (91.6)	617 (92.0)	91 (89.2)	0.354

Values are n (%) or median (Q1-Q3).

AF = atrial fibrillation; CLBBB = complete left bundle branch block; PPM = patient prosthesis mismatch; PVL = paravalvular leakage; other abbreviations as in Table 1.

In comparison with the SEV TAVI group, the BEV TAVI group more frequently underwent TAVI through the femoral approach (623/671 [93.0%] vs 90/102 [88.2%]; *P* < 0.001) and less frequently underwent TAVI using local anesthesia (221/671 [32.9%] vs 48/102 [47.1%]; *P* = 0.005). The older BEVs and SEVs were used in 99 of 671 (14.8%) cases in the BEV TAVI group and in 8 of 102 (7.8%) cases in the SEV TAVI group. The median valve size was 29 mm (range, 26-29 mm) in the entire cohort. Although the peak transaortic valve pressure gradient was higher in the BEV TAVI group than in the SEV TAVI group (18.5 mm Hg [range: 14.4-23.0 mm Hg] vs 16.0 mm Hg [range: 12.3-22.8 mm Hg]; *P* = 0.035), the incidence of moderate to severe PPM did not differ between the 2 groups.

The incidence of greater than moderate PVL was significantly higher in the SEV TAVI group (14/671 [2.1%] vs 8/102 [7.8%]; *P* < 0.001). Although the 2 groups showed no significant differences in the incidence of perioperative complications, the SEV TAVI group included a higher proportion of cases requiring a second valve (2/102 [2.0%] vs 3/671 [0.4%]; *P* = 0.076) and a significantly greater proportion of patients who required postoperative PMI (12/102 [11.8%] vs 41/671 [6.1%]; *P* = 0.035). However, the rates of technical and device success were comparable between the 2 groups. The complication rates for each device are shown in Supplemental Table 1. TAVI with the older devices was associated with the following: a lower rate of technical success; a significantly higher proportion of cases requiring a second valve, PMI, or conversion to surgery; and a significantly higher proportion of cases showing acute kidney injury.

In addition, in a previous paper, a large annulus area was defined as an extralarge annulus if the area was larger than 683 mm^2^ and as a large annulus if the area was larger than 575 mm^2^ but smaller than 683 mm^2^.^12^ The results of the analysis of the whole cohort of the present study divided into 3 groups according to this definition (group 1, annulus area ≤500 to <575 mm^2^; group 2, annulus area ≤575 mm^2^ to ≤683 mm^2^; group 3, annulus area >683 mm^2^) are shown in Supplemental Table 2. The number of patients with an extralarge annulus (area >683 mm^2^) was small (18 cases in total), and SEV was used less frequently in patients with an annulus area >575mm^2^. The incidence of PMI was lower in the BEV TAVI group, but there was no difference in PPM incidence or device success rate.

**Figure 1:**
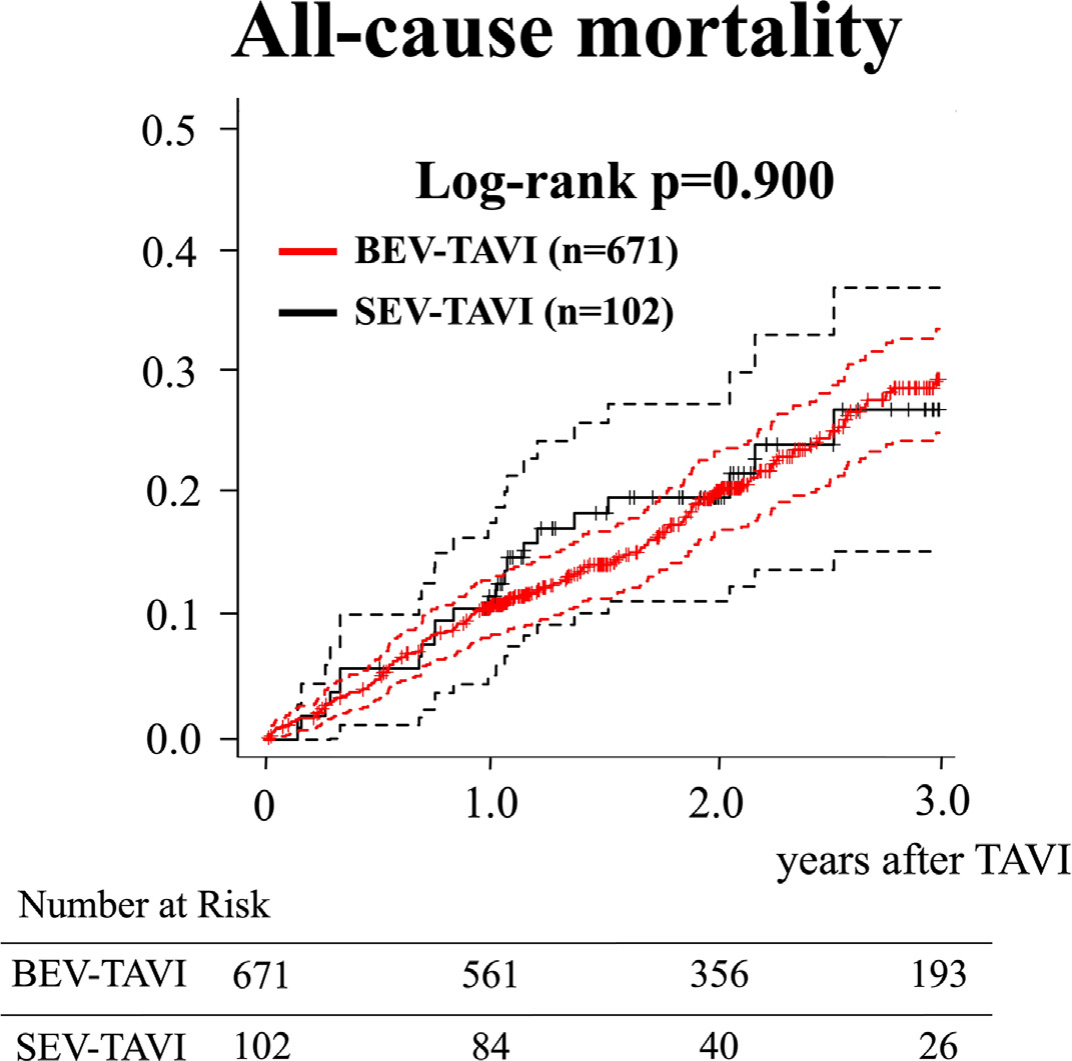
The Kaplan-Meier Curve for 3-Year Mortality After TAVI The dotted lines indicate 95% CIs. BEV = balloon-expandable valve; SEV = self-expandable valve; TAVI = transcatheter aortic valve implantation.

**Figure 2:**
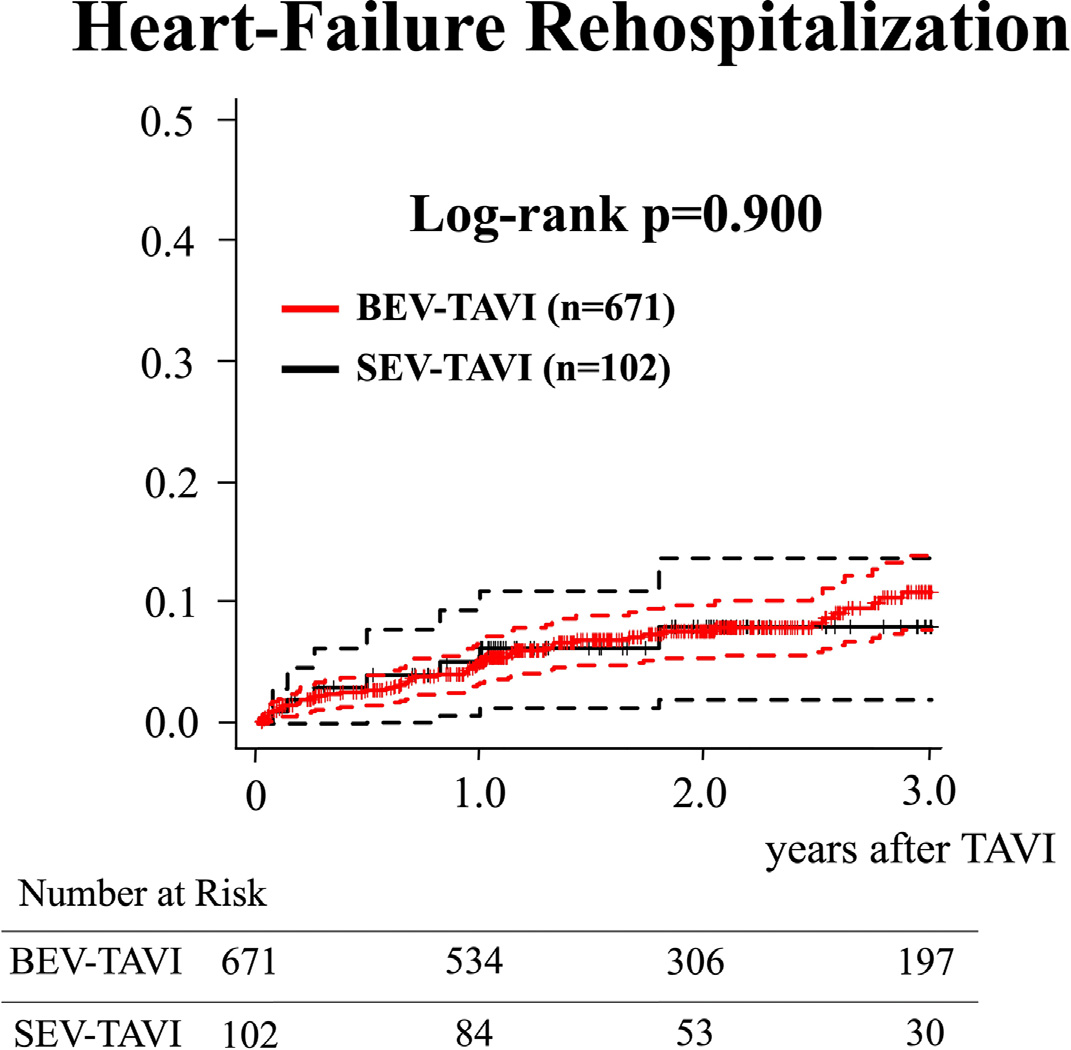
The Kaplan-Meier Curve for 3-Year Heart Failure Rehospitalization After TAVI The dotted lines indicate 95% CIs. Abbreviations as in Figure 1.

The Kaplan-Meier curve showed no significant differences in the 3-year all-cause mortality and HFR between the 2 groups (log-rank *P* = 0.900) (Figures 1 and 2). That is, all-cause mortality was 11.0% (95% CI: 8.6%-13.4%) vs 12.9% (95% CI: 6.1%-19.2%) at 1 year, 20.6% (95% CI: 17.2%-23.8%) vs. 22.0% (95% CI: 12.7%-30.4%) at 2 years, and 29.8% (95% CI: 25.4%-34.0%) vs 27.3% (95% CI: 15.6%-37.4%) at 3 years, and HFR rates were 5.3% (95% CI: 3.5%-7.0%) vs 6.2% (95% CI: 1.3%-11.0%) at 1 year, 7.9% (95% CI: 5.6%-10.2%) vs 8.0% (95% CI: 2.0%-13.7%) at 2 years, and 10.9% (95% CI: 7.7%-13.9%) vs 11.2% (95% CI: 4.8%-17.4%) at 3 years in the BEV TAVI and SEV TAVI groups, respectively.

**Figure 3:**
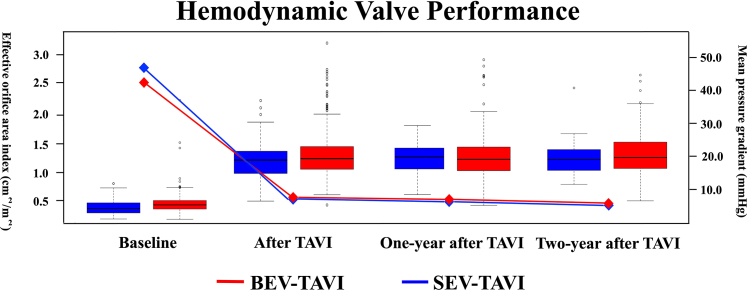
Hemodynamic Valve Performance After TAVI A boxplot represents the median effective orifice area index with a central line inside the box, the first and third quartiles with the lower and upper edges of the box, respectively, the minimum and maximum values with the ends of the whiskers, and outliers with individual points. The diamond-shaped dots indicate the mean aortic valve pressure gradient. Abbreviations as in Figure 1.

The echocardiography-indexed effective orifice area and mean transaortic valve pressure gradient 2 years after TAVI were not significantly different between the 2 groups (Figure 3). In addition, no cases of hemodynamic valve deterioration on the basis of the Valve Academic Research Consortium-3 criteria were observed during the 3-year postoperative period.

## Discussion

This is the largest study comparing BEV TAVI and SEV TAVI in patients with a large annulus, which was defined by an annular area of ≥500 mm^2^ and an average diameter of ≥25 mm on CT measurements, to report the short-term clinical outcomes and 3-year mortality and HFR rates after TAVI (Central Illustration). The main findings of this study are as follows:

1. Only 10.5% of patients had a large annulus in the largest Japanese TAVI registry, the OCEAN-TAVI registry cohort, and 86.8% of the patients with a large annulus underwent BEV TAVI.
2. The rates of technical and device success in the 2 groups were similar in the acute phase after TAVI; however, the rates of residual moderate or higher PVL and PMI were significantly higher in the SEV TAVI group.
3. The 3-year mortality rate, HFR rate after TAVI, and 2-year transcatheter heart valve performance rates were similar between the 2 groups.

**Central Illustration:**
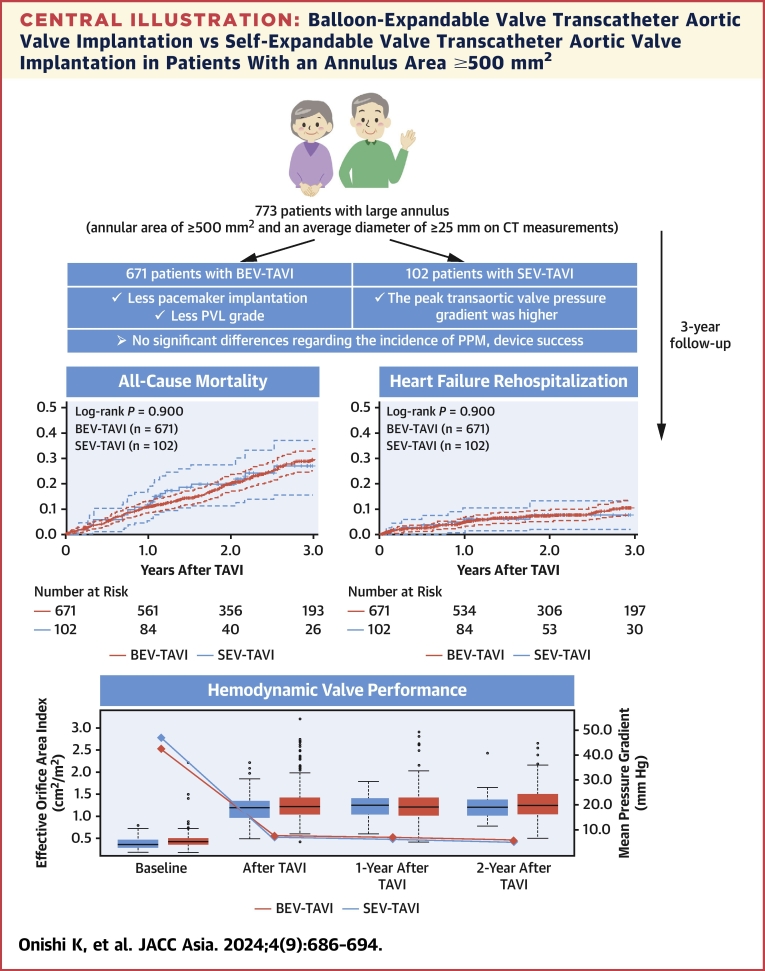
Balloon-Expandable Valve Transcatheter Aortic Valve Implantation vs Self-Expandable Valve Transcatheter Aortic Valve Implantation in Patients With an Annulus Area ≥500 mm^2^ This figure compares the short-term outcomes of self-expandable valve (SEV) transcatheter aortic valve implantation (TAVI) and balloon-expandable valve (BEV) transcatheter aortic valve implantation with all-cause mortality, heart failure rehospitalization at 3 years, and hemodynamic valve performances at 2 years after in patients with an annular area of ≥500 mm^2^ who underwent transcatheter aortic valve implantation. CT = computed tomography; PPM = prosthesis–patient mismatch; PVL = paravalvular leakage.

Although several studies have independently defined a large annulus and reported clinical outcomes comparing BEV TAVI and SEV TAVI, SEV TAVI has been reported to be associated with higher postoperative PVL grades and PMI rates in patients with a large annulus, similar to the findings of the present study.^9^, ^10^, ^11^, ^12^ Some reasons for the higher PMI rate and PVL grade in the SEV TAVI group may be the greater use of alternative access and the presence of bicuspid valves or left ventricular outflow tract calcification. Furthermore, it has been reported that TAVI results for patients with an extralarge annulus (area >683 mm^2^), especially patients with annulus areas beyond the recommended size range for the device, are poor given the higher incidence of second valve or conversion to open heart surgery.^17^ However, the small number of patients with an extralarge annulus in our study cohort makes it difficult to argue for similar results.

Analysis by valve type showed that the older SEV TAVI device was associated with a higher incidence of moderate or higher PVL, second valve requirement, and PMI, and it showed a lower technical success rate after TAVI. In contrast, the SEVs with a supra-annular design showed better valve performance after TAVI; however, no significant difference was observed in the incidence of moderate or severe PPM between the 2 groups. Furthermore, the incidence of severe PPM in the present study (BEV TAVI, 9/671 [1.3%]; SEV TAVI, 2/102 [2.0%]) was much lower than that in previous studies, which showed a lower frequency of PPM in patients with a large annulus than in patients with a small annulus.^9^^,^^10^ As mentioned in previous reports, this may reflect the fact that the patients enrolled in the OCEAN-TAVI registry, our study cohort, were Japanese and had a smaller body surface area than individuals from Western countries.^18^ In addition, although SEV TAVI is generally considered to be associated with a lower frequency of PPM than BEV TAVI, the hemodynamic advantages of SEV TAVI fade with increasing annulus size.^8^^,^^18^

In contrast, although the frequency of moderate or greater PMI and PVL was higher in the SEV TAVI group, it did not affect clinical outcomes at 3 years after TAVI, with no differences in the 3-year all-cause mortality and HFR rates between the 2 groups. However, previous studies have shown that greater PVL could be associated with mortality and HFR after TAVI.^19^, ^20^, ^21^ Therefore, the longer observation period in this study cohort may have produced differences in mortality and HFR.

The results of this study suggest that, in patients with a large annulus, the advantage of SEV TAVI in achieving better hemodynamics is limited, considering the frequency of postoperative PPM, and that BEV TAVI is superior to SEV TAVI in terms of the degree of PVL and the PMI rate.

### Study limitations

First, the criteria used to define a large annulus (annular area ≥500 mm^2^ and average diameter ≥25 mm on CT measurements) differed from those used in previous studies. However, this definition was based on the anticipated use of BEVs >26 mm and SEVs >29 mm because the average valve ring size in Japanese patients is small. The second limitation is the low percentage of SEV TAVI in all cohorts, with none of the patients receiving the 34-mm Evolut prosthesis during the enrollment period of this study. The third limitation of this study is that this was not a randomized controlled trial but a multicenter observational study, so the choice of valves was left to the judgment of the heart team members at each institution. Finally, long-term echocardiographic data are needed for detailed assessment of the incidence of hemodynamic valve deterioration after TAVI.

## Conclusions

The lack of differences in postoperative valve performance and long-term prognosis between BEV TAVI and SEV TAVI highlights the importance of selecting valves that can reduce the PMI rate and PVL grade in the acute phase after TAVI in patients with a large annulus.

Perspectives **COMPETENCY IN SYSTEMS-BASED PRACTICE:** In Japanese patients with a large annulus who undergo TAVI, SEV TAVI offers a limited hemodynamic advantage over BEV TAVI for a large annulus, whereas BEV TAVI is still superior in terms of the degree of PMI and PVL, as well as the incidence of ischemic stroke. Conversely, the prognosis at 3 years after TAVI did not differ between patients undergoing BEV TAVI and SEV TAVI.

**TRANSLATIONAL OUTLOOK:** It is important to select and treat valves to improve short-term outcomes in patients with a large annulus. However, whether differences in short-term outcomes influence differences in long-term prognosis should continue to be studied.

## Funding Support and Author Disclosures

The OCEAN-TAVI registry was supported by Edwards Lifesciences, Medtronic, Boston Scientific, Abbott Medical, and Daiichi-Sankyo. Dr Mizutani has served as a clinical proctor for Edwards Lifesciences and Medtronic. Dr Ohno has served as a clinical proctor for Medtronic. Dr Yashima has served as a clinical proctor for Medtronic. Dr Naganuma has served as a clinical proctor for Edwards Lifesciences and Medtronic. Dr Tada has served as a clinical proctor for Edwards Lifesciences and Medtronic. Dr Shirai has served as a clinical proctor for Edwards Lifesciences, Abbott Medical, and Medtronic. Dr Takagi has served as a clinical proctor for Edwards Lifesciences. Dr Asami has served as a clinical proctor for Medtronic. Dr H. Ueno has served as a clinical proctor for Edwards Lifesciences and Medtronic. Dr Watanabe has served as a clinical proctor for Edwards Lifesciences and Medtronic. Dr Yamamoto has served as a clinical proctor for Edwards Lifesciences, Abbott Medical, and Medtronic. Dr Hayashida has served as a clinical proctor for Edwards Lifesciences, Abbott Medical, and Medtronic. All other authors have reported that they have no relationships relevant to the contents of this paper to disclose.
