# Supplementary material for: Transcatheter Aortic Valve Implantation in Japanese Patients With Large Annulus: The OCEAN-TAVI Registry
Source: JACC Asia. 2024 Aug 13;4(9):686–94. doi: 10.1016/j.jacasi.2024.07.002 (PMC11450954; doi:10.1016/j.jacasi.2024.07.002)
Supplement: Supplemental Tables [file mmc1.docx]

| Parameters | Total  n=773 | SAPIEN XT  n=99 | SAPIEN 3  n=572 | CoreValve  n=8 | Evolut R  n=55 | Evolut Pro  n=39 | p-value |
| --- | --- | --- | --- | --- | --- | --- | --- |
| **Procedural Data** |  |  |  |  |  |  |  |
| Femoral approach | 713 (92.4%) | 65 (65.7%) | 558 (97.7%) | 7 (87.5%) | 46 (83.6%) | 37 (94.9%) | <0.001 |
| Local anesthesia | 269 (34.8%) | 8 (8.1%) | 213 (37.2%) | 1 (12.5%) | 27 (49.1%) | 20 (51.3%) | <0.001 |
| Puncture | 610 (80.3%) | 28 (29.5%) | 497 (88.0%) | 7 (87.5%) | 42 (79.2%) | 36 (92.3%) | <0.001 |
| Valve size (mm) | 29 (26-29) | 29 (26-29) | 26 (26-29) | 29 (29-29) | 29 (29-29) | 29 (29-29) | <0.001 |
| Procedure time (min) | 63 (49-85) | 90 (63-118) | 60 (45-76) | 61 (53-85) | 77 (63-106) | 69 (61-92) | <0.001 |
| **TTE Data after TAVI** |  |  |  |  |  |  |  |
| Mean AVPG (mmHg) | 9.0 (7.0-12.0) | 8.0 (5.6-9.4) | 9.8 (7.8-12.0) | 9.3 (7.4-13.0) | 8.2 (5.9-12.2) | 8.0 (7.4-9.3) | <0.001 |
| Peak AVPG (mmHg) | 18.1 (14.4-23.0) | 14.5 (11.0-18.9) | 19.4 (15.2-24.1) | 17.6 (9.7-26.1) | 17.3 (12.8-25.4) | 16.0 (12.3-20.1) | <0.001 |
| Effective orifice area index (cm^2^/m^2^) | 1.22 (1.04-1.43) | 1.35 (1.10-1.67) | 1.21 (1.03-1.41) | 1.18 (0.93-1.32) | 1.21 (1.06-1.34) | 1.18 (0.90-1.38) | 0.002 |
| PVL grade ≧moderate | 22 (2.9%) | 1 (1.0%) | 13 (2.3%) | 1 (12.5%) | 4 (7.3%) | 3 (7.7%) | <0.001 |
| Moderate PPM | 49 (6.4%) | 5 (5.1%) | 35 (6.1%) | 2 (25.0%) | 3 (5.5%) | 4 (10.3%) | 0.192 |
| Severe PPM | 11 (1.4%) | 1 (1.0%) | 8 (1.4%) | 0 (0.0%) | 1 (1.8%) | 1 (2.6%) | 0.956 |
| **Periprocedural Complications** |  |  |  |  |  |  |  |
| Major vascular complication | 19 (2.5%) | 4 (4.0%) | 13 (2.3%) | 1 (12.5%) | 0 (0.0%) | 1 (2.6%) | 0.209 |
| Coronary occlusion | 7 (0.9%) | 2 (2.0%) | 4 (0.7%) | 0 (0.0%) | 1 (1.8%) | 0 (0.0%) | 0.630 |
| Aortic root rupture | 1 (0.1%) | 0 (0.0%) | 1 (0.2%) | 0 (0.0%) | 0 (0.0%) | 0 (0.0%) | 0.986 |
| Second Valve | 5 (0.7%) | 2 (2.0%) | 1 (0.2%) | 1 (12.5%) | 0 (0.0%) | 1 (2.6%) | <0.001 |
| Conversion to open surgery | 2 (0.3%) | 2 (2.0%) | 0 (0.0%) | 0 (0.0%) | 0 (0.0%) | 0 (0.0%) | 0.008 |
| Permanent pacemaker implantation | 53 (6.9%) | 10 (10.1%) | 31 (5.4%) | 2 (25.0%) | 6 (10.9%) | 4 (10.3%) | 0.045 |
| New onset of CLBBB | 150 (21.3%) | 16 (16.7%) | 109 (21.1%) | 3 (37.5%) | 13 (27.1%) | 26 (25.7%) | 0.425 |
| New onset of AF | 27 (3.5%) | 7 (7.1%) | 17 (3.0%) | 0 (0.0%) | 1 (1.9%) | 2 (5.3%) | 0.274 |
| Ischemic Stroke | 20 (2.6%) | 3 (3.0%) | 12 (2.1%) | 0 (0.0%) | 4 (7.3%) | 1 (2.6%) | 0.229 |
| Acute kidney injury | 74 (9.6%) | 22 (22.2%) | 42 (7.3%)) | 2 (25.0%) | 5 (9.1%) | 3 (7.7%) | <0.001 |
| In-hospital death | 7 (0.9%) | 2 (2.0%) | 5 (0.9%) | 0 (0.0%) | 0 (0.0%) | 0 (0.0%) | 0.679 |
| Technical success | 749 (97.0%) | 92 (92.9%) | 558 (97.6%) | 7 (87.5%) | 55 (100.0%) | 37 (94.9%) | 0.031 |
| Device success | 708 (91.6%) | 90 (90.9%) | 527 (92.1%) | 6 (75.0%) | 51 (92.7%) | 34 (87.2%) | 0.378 |

Supplementary Table1. Procedural and Periprocedural Data in each Valve

Categorical variables are shown as numbers (percentages) and continuous variables are shown as medians (25-75 percentiles).

| **Group** | **500mm^2^≤annulus area<575mm^2^**  **(n=596)** | | | **575mm^2^≤annulus area≤683mm^2^**  **(n=162)** | | | **annulus area>683 mm^2^**  **(n=18)** | | |
| --- | --- | --- | --- | --- | --- | --- | --- | --- | --- |
| Parameters | BEV  n=498 | SEV  n=94 | p-value | BEV  n=155 | SEV  n=7 | p-value | BEV  n=17 | SEV  n=1 | p-value |
| **Baseline Data** | | | | | | | | | |
| Age (years) | 83 (80-86) | 84 (79-87) | 0.566 | 83 (79-86) | 83 (83-85) | 0.821 | 81 (77-83) | 82 | 0.923 |
| Male | 430 (86.2%) | 75 (79.8%) | 0.110 | 139 (89.7%) | 7 (100.0%) | 0.371 | 16 (94.1%) | 1 (100.0%) | 0.803 |
| BSA (m^2^) | 1.60  (1.50-1.70) | 1.58  (1.40-1.70) | 0.004 | 1.60  (1.50-1.75) | 1.60  (1.53-1.65) | 0.694 | 1.60  (1.50-1.70) | 1.70 | 0.459 |
| Bicuspid valve | 46 (9.3%) | 13 (14.1%) | 0.153 | 28 (18.2%) | 5 (71.4%) | 0.001 | 4 (23.5%) | 0 (0.0%) | 0.582 |
| CT data before TAVI |  |  |  |  |  |  |  |  |  |
| Annulus area (mm^2^) | 529.0  (513.8-549.0) | 523.0  (512.0-549.0) | 0.017 | 602.0  (587.0-632.5) | 606.0  (584.0-608.5) | 0.636 | 700.0  (693.0-714.9) | 711.8 | 0.778 |
| Mean Annular diameter (mm) | 26.0  (25.6-26.4) | 25.8  (25.6-26.2) | 0.036 | 27.7  (27.4-28.4) | 27.8  (27.3-27.9) | 0.620 | 29.9  (29.7-30.2) | 30.1 | 0.699 |
| Annular perimeter (mm) | 83.1  (81.7-84.6) | 82.2  (81.5-83.6) | 0.002 | 88.5  (87.3-90.7) | 87.3  (87.1-87.9) | 0.180 | 94.9  (94.2-96.7) | 93.4 | 0.261 |
| Moderate or severe LVOT calcification | 15 (3.1%) | 12 (13.1%) | <0.001 | 6 (4.0%) | 0 (0.0%) | 0.218 | 1 (5.9%) | 0 (0.0%) | 0.900 |
| **Procedural Data** |  |  |  |  |  |  |  |  |  |
| Femoral approach | 460 (92.4%) | 82 (87.2%) | <0.001 | 147 (94.8%) | 7 (100.0%) | 0.827 | 16 (94.1%) | 1 (100.0%) | 0.803 |
| Local anesthesia | 167 (33.5%) | 47 (50.0%) | 0.002 | 49 (31.6%) | 1 (14.3%) | 0.332 | 5 (29.4%) | 0 (0.0%) | 0.523 |
| Puncture | 389 (79.2%) | 77 (83.7%) | 0.326 | 122 (79.7%) | 7 (100.0%) | 0.185 | 14 (87.5%) | 1 (100.0%) | 0.707 |
| Valve size (mm) | 26 (26-29) | 29 (29-29) | <0.001 | 29 (29-29) | 29 (29-29) | 0.756 | 29 (29-29) | 29 | 0.860 |
| Procedure time (min) | 61 (47-84) | 77 (61-101) | <0.001 | 64 (49-82) | 63 (59-74) | 0.771 | 62 (46-93) | 60 | 0.828 |
| **TTE Data**  **after TAVI** |  |  |  |  |  |  |  |  |  |
| Mean AVPG (mmHg) | 9.0  (7.0-12.0) | 8.8  (6.1-12.0) | 0.198 | 9.5  (7.7-12.0) | 6.0  (5.0-8.0) | 0.033 | 10.0 (7.0-10.7) | 10.0 | 0.923 |
| Peak AVPG (mmHg) | 18.2  (14.4-23.0) | 16.5  (12.9-23.0) | 0.100 | 19.4  (14.4-23.1) | 10.2  (9.8-16.3) | 0.047 | 17.6 (14.4-24.6) | 21.0 | 0.699 |
| Effective orifice area index (cm^2^/m^2^) | 1.22  (1.04-1.43) | 1.20  (1.00-1.34) | 0.136 | 1.25  (1.06-1.41) | 1.00  (0.94-1.38) | 0.287 | 1.35 (1.13-1.51) | 1.50 | 0.588 |
| PVL grade ≧moderate | 9 (1.8%) | 8 (8.5%) | <0.001 | 4 (2.6%) | 0 (0.0%) | 0.058 | 1 (5.9%) | 0 (0.0%) | 0.645 |
| Moderate PPM | 32 (6.4%) | 8 (8.5%) | 0.457 | 8 (5.2%) | 1 (14.3%) | 0.303 | 3 (5.5%) | 4 (10.3%) | 0.192 |
| Severe PPM | 6 (1.2%) | 2 (2.1%) | 0.476 | 3 (1.9%) | 0 (0.0%) | 0.710 | 0 (0.0%) | 0 (0.0%) | - |

Supplementary Table2

| **Periprocedural Complications** |  |  |  |  |  |  |  |  |  |
| --- | --- | --- | --- | --- | --- | --- | --- | --- | --- |
| Major vascular complication | 15 (2.0%) | 2 (2.1%) | 0.640 | 2 (1.3%) | 0 (0.0%) | 0.762 | 0 (0.0%) | 0 (0.0%) | - |
| Coronary occlusion | 6 (1.2%) | 1 (1.1%) | 0.909 | 0 (0.0%) | 0 (0.0%) | - | 0 (0.0%) | 0 (0.0%) | - |
| Aortic root rupture | 0 (0.0%) | 0 (0.0%) | - | 1 (0.6%) | 0 (0.0%) | 0.831 | 0 (0.0%) | 0 (0.0%) | - |
| Second Valve | 2 (0.4%) | 2 (2.1%) | 0.061 | 0 (0.0%) | 0 (0.0%) | - | 1 (5.9%) | 1 (2.6%) | 0.803 |
| Conversion to open surgery | 2 (0.4%) | 0 (0.4%) | 0.539 | 0 (0.0%) | 0 (0.0%) | - | 0 (0.0%) | 0 (0.0%) | - |
| Permanent pacemaker implantation | 29 (5.8%) | 11 (11.7%) | 0.037 | 12 (7.7%) | 1 (14.3%) | 0.533 | 0 (0.0%) | 0 (0.0%) | - |
| New onset of CLBBB | 94 (20.6%) | 24(28.9%) | 0.090 | 29 (20.6%) | 0 (0.0%) | 0.181 | 2 (13.3%) | 1 (100.0%) | 0.032 |
| New onset of AF | 20 (4.1%) | 3 (3.3%) | 0.716 | 4 (2.6%) | 0 (0.0%) | 0.666 | 0 (0.0%) | 0 (0.0%) | - |
| Ischemic Stroke | 11 (2.2%) | 5 (5.3%) | 0.087 | 3 (1.9%) | 0 (0.0%) | 0.710 | 1 (5.9%) | 0 (0.0%) | 0.803 |
| Acute kidney injury | 50 (10.0%) | 8 (8.5%) | 0.651 | 13 (8.4%)) | 2 (28.6%) | 0.072 | 1 (5.9%) | 0 (0.0%) | 0.803 |
| In-hospital death | 5 (1.0%) | 0 (0.0%) | 0.330 | 1 (0.6%) | 0 (0.0%) | 0.831 | 1 (5.9%) | 0 (0.0%) | 0.803 |
| Technical success | 481 (96.4%) | 91 (96.8%) | 0.841 | 153 (98.7%) | 7 (100.0%) | 0.762 | 16 (94.1%) | 1 (100.0%) | 0.803 |
| Device success | 461 (92.4%) | 83 (88.3%) | 0.187 | 142 (91.6%) | 7 (100.0%) | 0.424 | 14 (82.4%) | 1 (100.0%) | 0.645 |

Categorical variables are shown as numbers (percentages) and continuous variables are shown as medians (25-75 percentiles).
